# Assessing the impact of climate warming on tree species composition and distribution in the forest region of Northeast China

**DOI:** 10.3389/fpls.2024.1430025

**Published:** 2024-07-29

**Authors:** Yuanyuan Fu, Chang Liu, Hong S. He, Shaoqiang Wang, Lunche Wang, Zhijie Xie

**Affiliations:** ^1^ Hubei Key Laboratory of Regional Ecology and Environmental Change, School of Geography and Information Engineering, China University of Geosciences, Wuhan, China; ^2^ Hunan Key Laboratory of Remote Sensing of Ecological Environment in Dongting Lake Area, School of Geography and Information Engineering, China University of Geosciences, Wuhan, China; ^3^ School of Natural Resources, University of Missouri, Columbia, MO, United States

**Keywords:** LINKAGES 3.0, climate warming, aboveground biomass, tree species composition and distribution, forest region of Northeast China

## Abstract

Global climate change has markedly influenced the structure and distribution of mid-high-latitude forests. In the forest region of Northeast China, the magnitude of climate warming surpasses the global average, which presents immense challenges to the survival and habitat sustainability of dominant tree species. We predicted the potential changes in aboveground biomass, dominant tree species composition, and distribution in the forest region of Northeast China over the next century under different climatic conditions encompassing the current scenario and future scenarios (RCP2.6, RCP4.5, and RCP8.5). Forest ecosystem process model LINKAGES 3.0 was used to simulate dynamic changes in species-level aboveground biomass under four climate scenarios at the homogeneous land-type unit level. The potential spatial distribution of tree species was investigated based on three indicators: extinction, colonization, and persistence. The results showed that LINKAGES 3.0 model effectively simulated the aboveground biomass of 17 dominant tree species in the forest region of Northeast China, achieving a high accuracy with R² = 0.88. Under the current, RCP2.6, and RCP4.5 climate scenarios, the dominant tree species presented gradual increases in aboveground biomass, whereas under RCP8.5, an initial increase and subsequent decline were observed. With increasing warming magnitude, cold-temperate coniferous tree species will gradually be replaced by other temperate broad-leaved tree species. Furthermore, a large temperature increase under RCP8.5 will likely produce a significant contraction in the potential distribution range of tree species like Larch, Scotch pine, Ribbed birch, Spruce and Fir, while most temperate broad-leaved tree species and Korean pine are expected to demonstrate a northward migration. These findings provide guidance for enhancing the adaptability and resilience of forest ecosystems in middle and high latitudes and addressing the threats posed by climate warming.

## 1 Introduction

Global climate change is instigating extensive alterations in suitable habitats of forest species worldwide (Scheffers et al., 2016) and has far-reaching consequences for the distribution, composition, and functionality of typical forest ecosystems (Freeman et al., 2018; Liang et al., 2023). Forest ecosystems exhibit pronounced sensitivity to shifts in temperature and precipitation regimes (Marchi et al., 2018), and tree species found at middle and high latitudes are particularly susceptible to these impacts. Climate warming has caused distributional changes (e.g., northward migration or habitat degradation) and local extinctions of many forest species, thereby negatively affecting the biodiversity, stability, and sustainability of these ecosystems (Saenz-Romero et al., 2017; Garate-Escamilla et al., 2019). In this context, forest management encounters pressing and formidable challenges, demanding the implementation of scientifically informed strategies to ensure ecosystem stability and sustainability (Nelson et al., 2016), thus safeguarding essential services and maintaining stable carbon storage (Nunes et al., 2019; Alejandra et al., 2020). Hence, understanding and addressing the influences of climate warming on forest tree species composition and distribution are vital for maintaining essential ecosystem functions, promoting sustainable development, and engaging in prudent and adaptive forest management practices.

As a unique and essential component of the global temperate forest ecosystem (Li et al., 2019), the forest region of Northeast China contains China’s unique forest vegetation zones of cold temperate coniferous forests, temperate coniferous and broad-leaved mixed forests, and warm temperate deciduous broad-leaved forests (Fu et al., 2019), and it harbors characteristic tree species adapted to temperate and cold climate zones. In particular, the climate in Northeast China has undergone substantial changes, with temperature increases in recent decades surpassing global values (Xu et al., 2011). Significant climate warming has had definite influences on the distribution and composition of tree species in this particular region, including the migration of tree species towards northern and high-altitude areas (Li and Huang, 2022), degradation of tree habitats (Jian et al., 2022), and variations in the proportion of broad-leaved and coniferous species (Liu et al., 2020). Many studies have also predicted the potential spatial distribution of tree species in the forest region of Northeast China under various climate change scenarios, indicating that climate warming and succession will profoundly impact tree species, leading to northward shifts in forests (Cheng and Yan, 2007), changes in species composition (Luo et al., 2019), and the extinction of dominant species or their replacement by species better adapted to new climate conditions (Deng et al., 2000; Hao et al., 2001). Simulating tree species migration in the forest region of Northeast China is essential for comprehensively assessing climate change impacts across its entire ecosystem, providing crucial insights for regional policy formulation and management strategies tailored to diverse ecological and spatial dynamics.

Currently, multiple types of predictive models have been used to simulate and quantitatively assess potential changes in the composition and habitat of tree species in middle and high latitudes under climate warming (Vanderwel and Purves, 2014). Bioclimatic envelope models utilize multiple statistical techniques to investigate the intricate relationship linking the observed species distribution with environmental factors. Recent advancements in bioclimatic envelope models involve incorporating dispersal functions to predict species range shifts, considering factors such as seed source and dispersal distance (Wang et al., 2015). However, the dynamic processes of species tracking environmental niches have not been thoroughly addressed (Boulangeat et al., 2012; Notaro et al., 2012). Biophysical process models describe plants as a dynamic system that integrates multiple physiological processes, although they typically employ coarse spatial resolutions and overlook or simplify processes at the site or landscape scale (Purves and Pacala, 2008; Iverson et al., 2017). Although forest landscape models explicitly consider plot-scale evolution and landscape-scale disturbances, they have limitations in capturing dynamic responses to climate warming (Gustafson et al., 2010; Seidl et al., 2012). Most dynamic vegetation models simulate instantaneous changes or short-term eco-physiological processes in vegetation and ecosystem characteristics, typically parameterized for specific study locations or regions, and are rarely employed for predicting large-scale vegetation changes (Tang and Beckage, 2010). Spatially explicit patch occupancy models have also found application in simulating changes in tree species distribution; however, their descriptions of processes at the population level are highly simplified (Garcia-Valdes et al., 2013). Each of these approaches serves a specific application; however, none is sufficiently appropriate for assessing potential changes in regional-scale forest distribution within the context of climate warming. The development of the LINKAGES 3.0 model offers a promising solution by integrating physiological mechanisms and allowing the simulation of time-evolving dynamics, shifts in forest structure, and alterations in species composition under varying climatic conditions.

LINKAGES, originating from gap models, is a plot-based forest ecosystem process model (Wullschleger et al., 2003), and it was revised by Dijak et al. in 2017 to version 3.0. Forest gap models have shown their competence in effectively simulating population dynamics and the repercussions of disturbances within intricate and multi-species forest stands (Yan and Shugart, 2005; Cheng et al., 2014). The model intricately links biological processes with non-biological processes by predicting decomposition processes, actual evapotranspiration, soil water balance, nutrient intake, tree growth, and light penetration through the canopy using a system of equations (Pastor and Post, 1986). The LINKAGES 3.0 model demonstrates notable strengths in simulating tree species composition and distribution at large regional scales and across multiple tree species, and it has been extensively and successfully applied in temperate zones, such as forests in the United States (Wang et al., 2017, 2019). Although previous studies have applied the LINAKGES model to elucidate the dynamics underlying diverse tree species in the forest region of Northeast China (Liang et al., 2011; Huang et al., 2021), they have predominantly focused on specific forest regions and tree species without comprehensively utilizing forest ecosystem processes and climate models to investigate the potential distribution of all dominant tree species throughout the forest region of Northeast China under changing climatic conditions.

The objective of this study was to investigate the spatiotemporal changes of tree species composition and distribution across the entire forest region of Northeast China under climate warming. Specifically, we utilized the forest ecosystem process model LINKAGES 3.0 to simulate the dynamic changes in aboveground biomass of 17 dominant tree species, and then based on the simulated biomass of each tree species, we analyzed the dynamics of tree species composition, and forecasted the potential spatial distribution of these species under current and future climate warming scenarios (optimistic-RCP2.6, moderate-RCP4.5, and pessimistic-RCP8.5) over the next 100 years in the forest region of Northeast China, starting from the year 2000. Three pivotal indicators (extinction, colonization, and persistence) were employed to elucidate the potential spatial distribution of dominant tree species in response to a warming climate. We hypothesized that the interaction between climate change and succession would be the primary driver of variation in aboveground biomass and species distribution, while climate change would account for the majority of changes in the distribution of some species by 2100. We hypothesized that with increasing warming magnitude, cold-temperate coniferous tree species will gradually be replaced by other temperate broad-leaved tree species. Through a comparative analysis of diverse climate scenarios and their consequent impacts on forest dynamics, this study aimed to reveal the response and feedback mechanisms to climate warming of forest ecosystems across the entire Northeast China, and thus provide theoretical guidance and reference support for forest resource management and decision-making in the region.

## 2 Materials and methods

### 2.1 Study area

**Figure 1:**
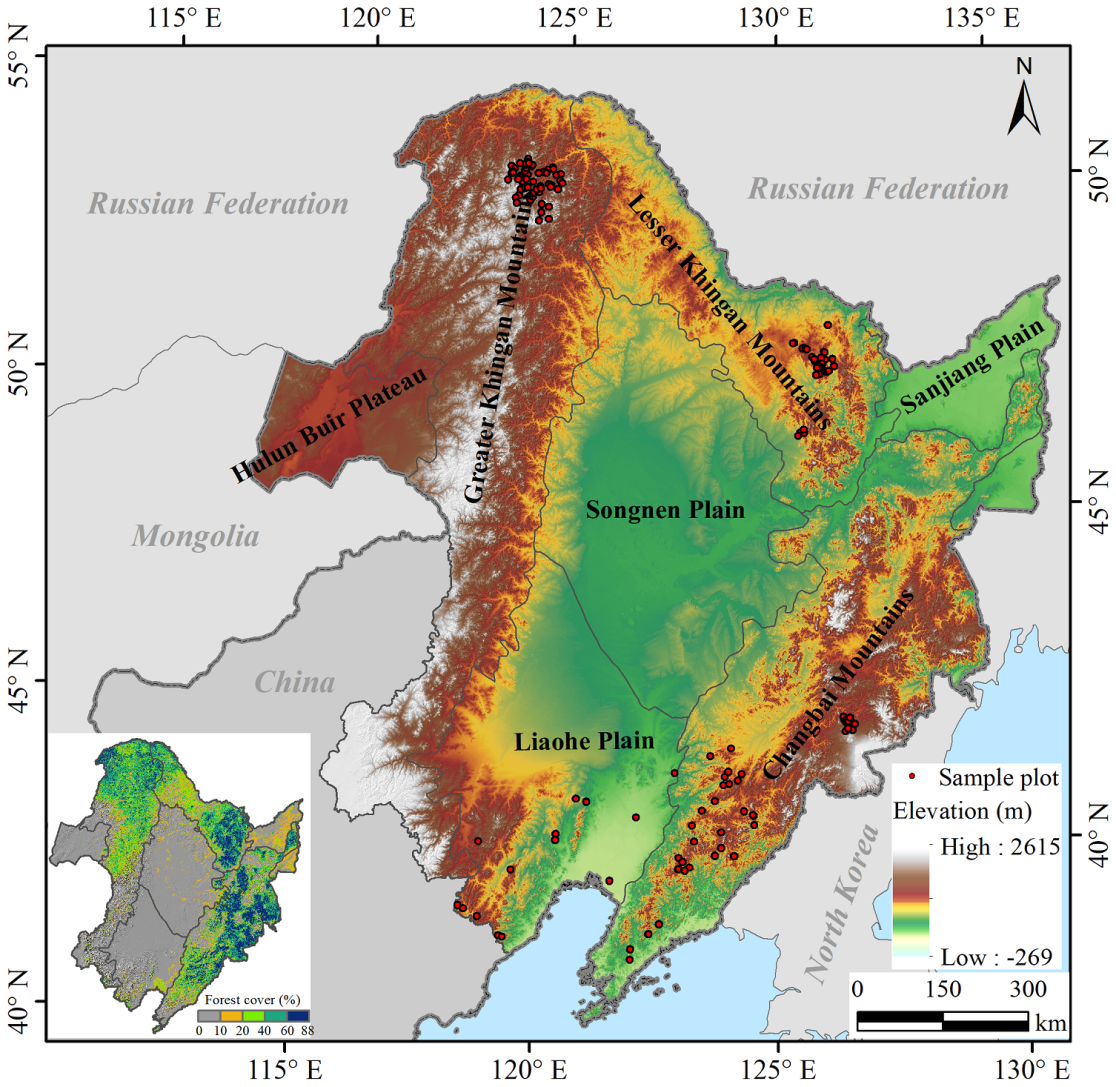
Spatial distribution of the seven subregions, elevation, sample plots, and forest cover fraction in Northeast China. The forest cover fraction was derived from the Vegetation Continuous Fields (VCF) product MOD44B (250 m) of Moderate Resolution Imaging Spectroradiometer (MODIS) images.

Our study area is situated in northeastern China (ranging from 38°42′N, 115°32′E to 53°35′N, 135°09′E) and encompasses a substantial coverage of 124 million hectares. This expansive territory encapsulates the provinces of Heilongjiang, Jilin, and Liaoning, as well as the eastern Inner Mongolia Autonomous Region (**Figure 1**). This region has diverse climates, soils, vegetation types, and terrains over an elevation range of -269 to 2,615 m, and it is characterized by seven subregions (Fu et al., 2019): Greater Khingan Mountains (GKM), Lesser Khingan Mountains (LKM), Changbai Mountains (CBM), Sanjiang Plain (SJP), Songnen Plain (SNP), Liaohe Plain (LHP), and Hulun Buir Plateau (HBP) (**Figure 1**). The study area has a humid and semi-humid temperate continental monsoon climate, which features hot and rainy summer alongside cold and dry winter. The average annual temperature and precipitation range from -7.0 to 12.4°C and from 230.6 to 1032.8 mm, respectively (Du et al., 2013).

Roughly 40% of the area is encompassed by forests (Pan et al., 2011), fostering an abundant variety of tree species and an assortment of forest types (Xiao et al., 2002), which are primarily concentrated across the subregions of the GKM, LKM, and CBM (**Figure 1**). Temperate cool coniferous forests dominate the northernmost part of the GKM, while temperate coniferous and broad-leaved mixed forests dominate the LKM and CBM. The major species include deciduous conifers, such as Larch (*Larix gmelinii* (Rupr.) Kuzen); evergreen conifers, such as Spruce (*Picea koraiensis* Nakai), Korean pine (*Pinus koraiensis* Sieb.et Zucc.), Mongolian Scotch pine *(Pinus sylvestris* var. *mongolica* Litv., hereafter Scotch pine), and Fir *(Abies nephrolepis* (Trautv.) Maxim.); and deciduous broad-leaved species, such as White birch (*Betula platyphylla* Suk.), Aspen (*Populus davidiana* Dode.), Mongolian oak *(Quercus mongolica* Fisch. ex Ledeb.), Asian Black birch (*Betula davurica* Pall.; hereafter, black birch), Ribbed birch (*Betula costata* Trautv.), Basswood *(Tilia amurensis* Rupr.), Mono maple (*Acer mono* Maxim.), Elm (*Ulmus pumila* L.), Manchurian Walnut *(Juglans mandshurica* Maxim.; hereafter, walnut), Manchurian Ash (*Fraxinus mandschurica* Rupr.; hereafter ash), Amur corktree (*Phellodendron amurense* Rupr.), and Willow (*Chosenia arbutifolia* (Pall.) A. Skv.).

### 2.2 Climate warming scenarios and climate data

We integrated the current climate as a baseline climate scenario and introduced three climate warming scenarios utilizing the MRI-CGCM3 general circulation model (GCM), which corresponds to three representative concentration pathways (RCPs): 2.6, 4.5, and 8.5. These scenarios are expressed through RCPs, representing radiative forcing levels between the preindustrial era and 2100 (IPCC, 2013). Specifically, the optimistic scenario (RCP2.6) assumes a 2100 CO_2_ concentration of 450 ppm, resulting in a global mean temperature increase of 0.2-1.8°C. The moderate (RCP4.5) and pessimistic (RCP8.5) scenarios anticipate a 650 ppm and 1350 ppm CO_2_ concentration, respectively, with temperature increase of 1.0-2.6°C and 2.6-4.8°C by 2100. The MRI-CGCM3 model with fine spatial resolution (1.125°×1.125°), which was crafted by the Meteorological Research Institute (MRI) in Japan, credibly simulates historical climates and performs well in predicting future climatic conditions over Northeast China (Sun et al., 2015; Lu et al., 2020). The three emission scenarios (RCP 2.6, RCP 4.5, and RCP 8.5) of the MRI-CGCM3 model effectively capture the warming trends and annual variations in precipitation for future climate projections in our research zone (Meinshausen et al., 2011).

Daily climate data (including maximum and minimum temperatures, precipitation, mean surface wind speed, and incident solar radiation) for the current climate scenario (1980–2015) were obtained from the China Meteorological Data Service Center (http://data.cma.cn). The daily climate projection data for MRI-CGCM3 (2006–2100) were sourced from the Coupled Model Intercomparison Project Phase 5 (CMIP5, https://esgf-node.llnl.gov/projects/cmip5/). The current climate data was interpolation to 0.5°× 0.5° grids, with topographic effects incorporated through collaborative Kriging interpolation methods implemented based on MATLAB software. Future climate data was downscaled to the same grid resolution of 0.5°× 0.5°, leveraging the interpolated current climate data at a finer scale (Wang and Chen, 2014).

### 2.3 LINKAGES 3.0 model and parameterization

We used the species-specific forest ecosystem process model LINKAGES 3.0 to simulate the forest dynamics and the growth potential of tree species in response to climate change at regional scales. LINKAGES 3.0 was designed to simulate the intricate processes of forest succession and soil nutrient cycling by linking the interactions of biotic (regeneration, growth, and mortality) and abiotic processes (temperature and moisture regulation) through equations that predict tree growth, soil water balance, nutrient uptake, decomposition processes, actual evapotranspiration, and light penetration through the canopy (Dijak et al., 2017), the simulation results of which mainly include the number of standing trees, tree species biomass, litter production, net primary productivity, soil organic matter content, and basal area. In detail, the LINKAGES 3.0 model is comprised of seven distinct submodules: the Temperature submodule (TEMPE), Moisture submodule (MOIST), Soil Decomposition submodule (DECOMP), Accumulation submodule (GMULT), Tree Regeneration submodule (BIRTH), Growth submodule (GROW), and Mortality submodule (KILL). Among these, the Tree Regeneration, Growth, and Mortality submodules systematically calculate the dynamics of forest population based on the growth status of individual trees. Climatic conditions and soil characteristics are key factors that determine the regeneration of individual trees, biomass accumulation, stand dynamics, and soil carbon-nitrogen cycling (Dale et al., 2009). By adjusting the climatic parameters, the potential reactions of tree species distribution to climate change can be predicted.

The forest region of Northeast China was partitioned into 769 homogeneous land type units with identical ecological conditions based on terrain, altitude, climate, vegetation, and soil type, and each land type representing a standardized homogenous ecological condition within the area. The LINKAGES 3.0 model was then utilized to simulate the potential distribution of the 17 dominant tree species within the region across distinct climate warming scenarios at the level of individual land type. The 17 dominant tree species in the entire forest region of Northeast China were identified by analyzing the species composition of 25,000 forest stand polygons in the early 2000s collected from the National Forestry and Grassland Science Data Center (http://www.cfsdc.org/). The model parameters were categorized into four main types: climate, soil, tree species, and forest stand. The climate parameters were based on data from both current and projected climate warming scenarios. Soil parameters, including soil layer number, thickness, and sand-gravel ratio, were obtained from the Chinese Soil Science Database (http://vdb3.soil.csdb.cn/) and adjusted based on soil records and data from the National Forestry and Grassland Science Data Center (http://www.cfsdc.org/). Tree species parameters were obtained from the China Plant Species Information Database (http://db.kib.ac.cn/) and previous research conducted in the study region (Liang et al., 2014; Huang et al., 2021). The comprehensive details of these parameters, essential for understanding the unique characteristics of each of the 17 species under consideration, are meticulously presented in the supplementary table (**Supplementary Table S1**). The LINKAGES 3.0 model requires stand information for each land type in 2000 to ensure an accurate reflection of the initialized stand status, mainly including the serial number of tree species, the number of standing trees of each species, the diameter at breast height, slope and aspect. Our study employed species distribution density information in different diameter classes in each land type over the forest region of Northeast China in 2000, as estimated by Fu et al. (2022), combined with topographic data (slope and aspect) derived from the Shuttle Radar Topography Mission (SRTM) digital elevation model (DEM) with a 90-m spatial resolution (http://srtm.csi.cgiar.org/), to initialize stand parameters.

### 2.4 Experimental design and data analysis

We designed a single-factor experiment with four levels of climate, including the current climate scenario and three climate warming scenarios (RCP2.6, RCP4.5, and RCP8.5). We employed LINKAGES 3.0 model to predict the potential distribution changes of 17 dominant tree species in the forest region of Northeast China from 2000 to 2100 at a 1-year time step, with uniform initial forest conditions applied across all four simulation scenarios. To capture stochastic variations, we performed three replicate simulations for each climate scenario; however, only one replicate simulation result per scenario was randomly selected and reported because of minimal differences among the replicated simulations (Wang et al., 2019). The current climate scenario was treated as the baseline scenario.

We summarized the differences in species-level aboveground biomass among four scenarios, and mapped the percentage changes in total aboveground biomass for each land type under given climate warming scenario to current climate scenario at year 2040, 2070, and 2100 to represent short-, medium-, and long-term responses, respectively. Species occurrence was calculated as the percentage of the total land types in which a species was present. Specifically, we summarized the percentage variation in species occurrence under four climate scenarios in year 2040, 2070, and 2100 in Northeast China to help characterize the changes of species potential distribution. In addition, to investigate the projected tree species migration caused by climate warming, we examined and mapped the extinction, colonization, and persistence results under three climate warming scenarios in the forest region of Northeast China at year 2040, 2070, and 2100 relative to the current climate scenario. The three indicators of extinction, colonization, and persistence were interpreted as changes in species from present to absent, absent to present, or remained present within each land type, respectively (Wang et al., 2017). Furthermore, we compiled a summary of the proportions of colonization, extinction, and persistence across the entire study area.

A repeated-measures analysis of variance was used in this study to assess the relative influence of climate scenarios, years (i.e., succession), and their interaction on both the aboveground biomass and distribution of tree species. The response variable was the percent occurrence of each of the 17 tree species within the study area based on data collected from three replicates at 5-year intervals from 2000 to 2100 under each of the four climate scenarios. We determined the contribution of each factor and their interactions to the total variability of the response variable using Type III sum of squares and expressed the results as percentages. To account for the repeated-measures design, we also included *P* values to indicate the statistical significance of each factor.

### 2.5 Model validation

The data collected from the forest inventory plots were utilized to validate the LINKAGES 3.0 model. We selected detailed survey data collected from 165 sample plots distributed within the three major forest regions of Northeast China (Greater Khingan Mountains, Lesser Khingan Mountains, and Changbai Mountains) (**Figure 1**) to evaluate the simulated results from LINKAGES 3.0 model for the year 2011. Each sample plot (20 m × 30 m) recorded GPS coordinates, slope, aspect, elevation, forest type, and dominant species. Furthermore, the diameter at breast height (DBH), and tree height of every tree with a DBH larger than 5 cm were measured for each sample plot. Specifically, 165 sample plots data were used to estimate observed aboveground biomass for each tree species by applying species-specific biomass equations (Dong et al., 2014, 2015) based on the number of individuals in various diameter classes in each plot. Then we extracted the species-level aboveground biomass in 2011 simulated by LINKSGES 3.0 model under current climate at the corresponding 165 plot locations. Subsequently, we validated the overall accuracy of the LINKAGES 3.0 model by fitting simulated and observed total aboveground biomass for all tree species across 165 sample plots, and assessed its applicability to individual tree species by comparing simulated and observed total aboveground biomass values for each species across all plots.

## 3 Results

### 3.1 Model validation

**Figure 2:**
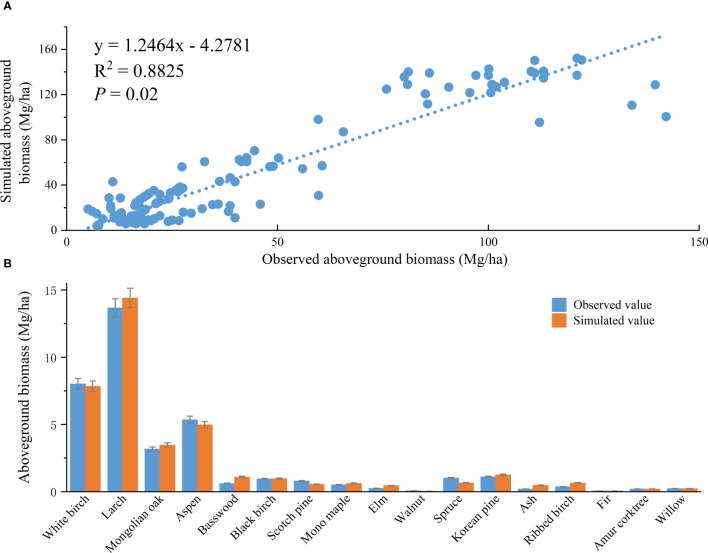
Validation of the model-simulated results based on observations of the **(A)** total aboveground biomass and **(B)** aboveground biomass of individual tree species. Error bars represent the standard deviation of the data.

From a comprehensive perspective, the simulated aboveground biomass generated through the LINKAGES 3.0 model at the plot level exhibited a discernible congruence with the surveyed values acquired from the forest inventory data (**Figure 2**). A strong and statistically significant correlation was identified between the simulated and observed aboveground biomass (R^2^ = 0.88), suggesting that the LINKAGES 3.0 model can accurately simulate the total aboveground biomass of 17 tree species in the forest region of Northeast China. Additionally, an analysis of biomass data from 165 surveyed sample plots and simulations conducted using LINKAGES 3.0 showed that the simulated biomass of most tree species closely matched the observed values, with no significant discrepancies identified at *P* < 0.05. This finding further confirms the reliability of the simulated aboveground biomass not only for the total aboveground biomass but also for individual tree species in the forest region of Northeast China.

### 3.2 Future climate change

**Figure 3:**
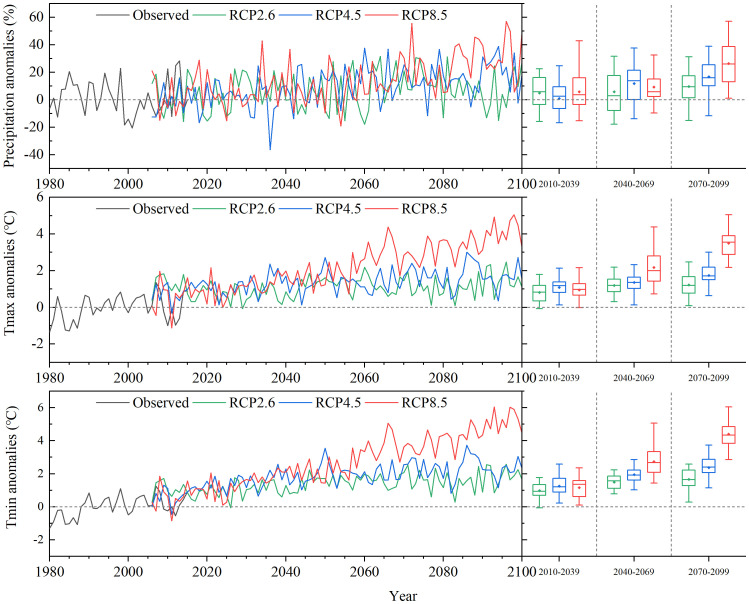
Temporal evolution curve of anomalies in (top to bottom) annual mean precipitation percentage (units: %), annual average air maximum temperature (Tmax), and air minimum temperature (Tmin) (units: °C) under the observed (black), RCP2.6 (green), RCP4.5 (blue), and RCP8.5 (red) scenarios over Northeast China during 1980 to 2100. The three groups of boxplots on the right reflect the characteristics (including maximum, minimum, median, and mean value) of the anomalies under the three RCPs in the early (2010–2039), mid (2040–2069), and end (2070–2099) periods of the 21st century.

The temporal evolution sequences of precipitation, Tmax, and Tmin projected by the MRI-CGCM3 model under the three RCPs scenarios in Northeast China represented anomalies relative to the observed data from 1986 to 2015. Overall, the predicted values of precipitation, Tmax, and Tmin in the 21st century showed a continuous increase, regardless of the emission scenario (**Figure 3**). In contrast to the baseline climate scenario, the annual mean precipitation under RCP2.6, RCP4.5, and RCP8.5 increased by 8.04%, 15.20%, and 24.79% in the end-21st century, respectively. From the baseline period to the end-century period (2070–2099), Tmax showed sustained increases across all three climate warming scenarios (RCP2.6, RCP4.5, and RCP8.5), with eventual respective elevations at 1.24°C, 1.77°C, and 3.51°C, respectively. Compared with the sharp rise observed for the RCP8.5 scenario over time, the trends for RCP2.6 and RCP4.5 were relatively gradual, with RCP4.5 showing a more pronounced increase in Tmax relative to RCP2.6 only by the end-century period (2070–2099). During the 21st century, the overall trends and data distribution characteristics of the simulated Tmin values across the three RCP scenarios were essentially consistent with those of Tmax. Notably, the increasing trend of Tmin was greater than that of Tmax throughout each period.

**Figure 4:**
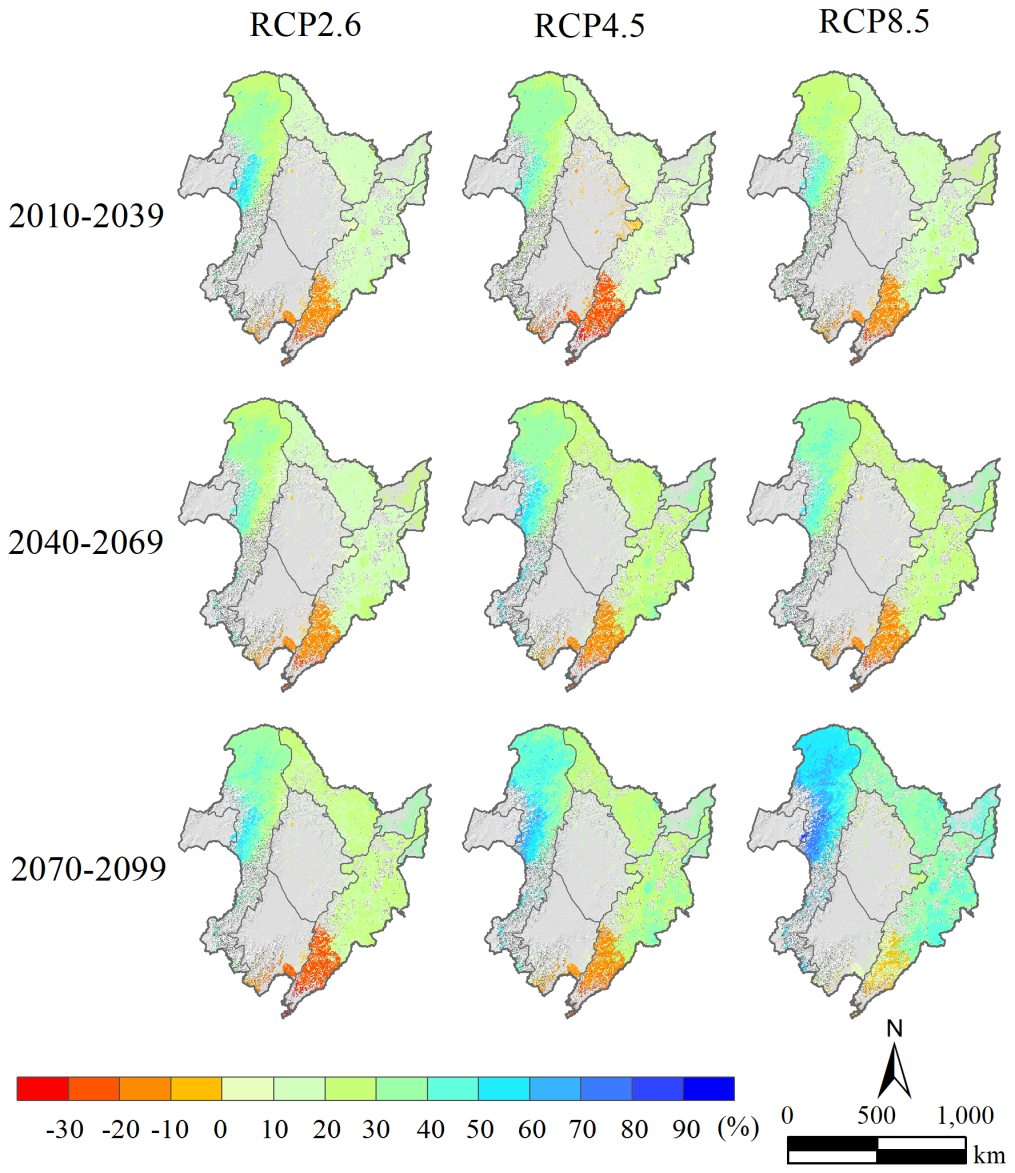
Spatial distributions of annual average precipitation percentage variations in the forest region of Northeast China during the early (2010–2039), mid (2040–2069), and end (2070–2099) 21st century intervals under the RCP2.6, RCP4.5, and RCP8.5 scenarios relative to the 1980–2009 period (units: %).

**Figure 5:**
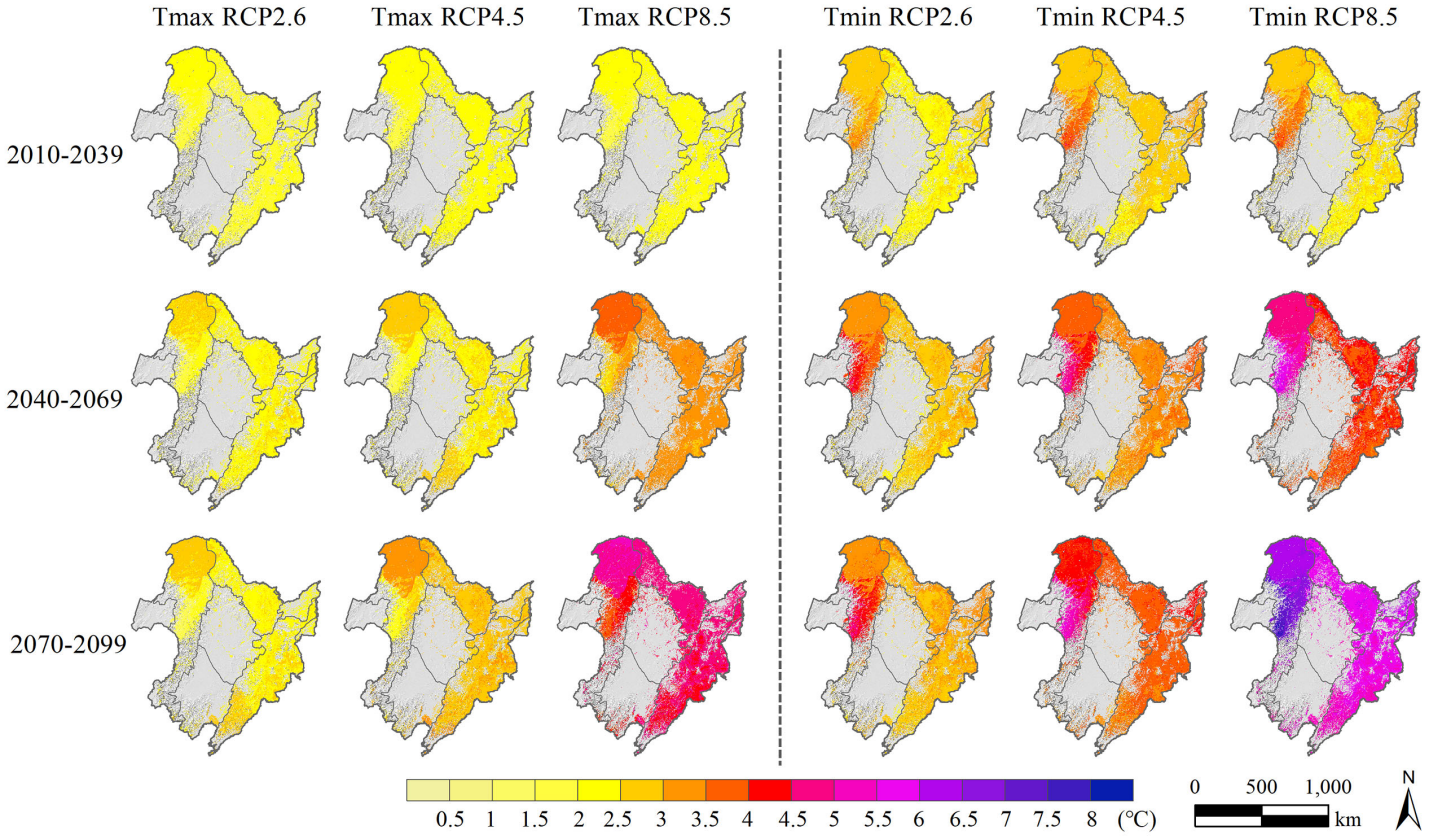
Spatial distributions of the annual mean air maximum temperature (Tmax) and air minimum temperature (Tmin) variations in the forest region of Northeast China during the early (2010–2039), mid (2040–2069), and end (2070–2099) 21st century intervals under the RCP2.6, RCP4.5, and RCP8.5 scenarios relative to the 1980–2009 period (units: °C).

The research findings demonstrate that the spatial patterns of precipitation, Tmax, and Tmin differ significantly across Northeast China, with changes of varying magnitudes observed among the various emission scenarios and time periods (**Figures 4**, **5**). For the annual mean precipitation, during the entire prediction period (2010–2099), the forest region of Northeast China showed an increasing trend in almost all areas under the three distinct climate warming scenarios (RCP2.6, RCP4.5, and RCP8.5), except in the southern part of the CBM, where precipitation decreased significantly relative to the baseline period (**Figure 4**). According to the predictions, the annual mean precipitation increase in the GKM (more than 30% in most areas) was expected to be significantly higher than that in the LKM and CBM (less than 30% in most areas) throughout all periods considered, with a substantially greater magnitude occurring in the middle-western area of the GKM (more than 40%). In general, the annual mean precipitation in the 21st century exhibited a significant increasing trend compared to the current climate (excluding the southern CBM), with the largest increase occurring in the GKM.

During the early-21st century (2010–2039), the simulated annual Tmax in the forest region of Northeast China exhibited a spatially homogenous increasing trend, with limited discernible differences among the three emission scenarios (**Figure 5**). By the mid-century (2040–2069), the annual Tmax in the study area had risen further from the previous period, with the largest increase observed in the northern GKM. Furthermore, a noteworthy increase in Tmax values was observed by the end-century period (2070–2099) in nearly all pixels, with a further widening of the gap in Tmax among the different scenarios. RCP8.5 exhibited the highest Tmax values (more than 4.0°C in most areas), followed by RCP4.5 (more than 2.5°C in most areas), with RCP2.6 presenting the lowest Tmax values (more than 2.0°C in most areas). In comparison to baseline period, the northern region of the GKM showed the greatest amplitude of Tmax increase, while the central region experienced relatively weak warming. A warming trend was also evident in the LKM and CBM, with the magnitude of warming falling between that of the north and central regions of the GKM. Unlike Tmax, the central region of the GKM demonstrated the largest increase in Tmin during the entire projected period from 2010 to 2099 under the three climate warming scenarios. By the mid-century (2040–2069), significant differences in the increase in Tmin were evident among the three climate scenarios and continued until the end 21st century. Overall, the most pronounced rise in Tmin within the forest region of Northeast China occurred in the central GKM, followed by the northern region of the GKM, while the LKM and CBM displayed similar spatial patterns of Tmin increase but with a less extensive magnitude compared to that in the GKM.

### 3.3 Changes in aboveground biomass and tree species composition

**Figure 6:**
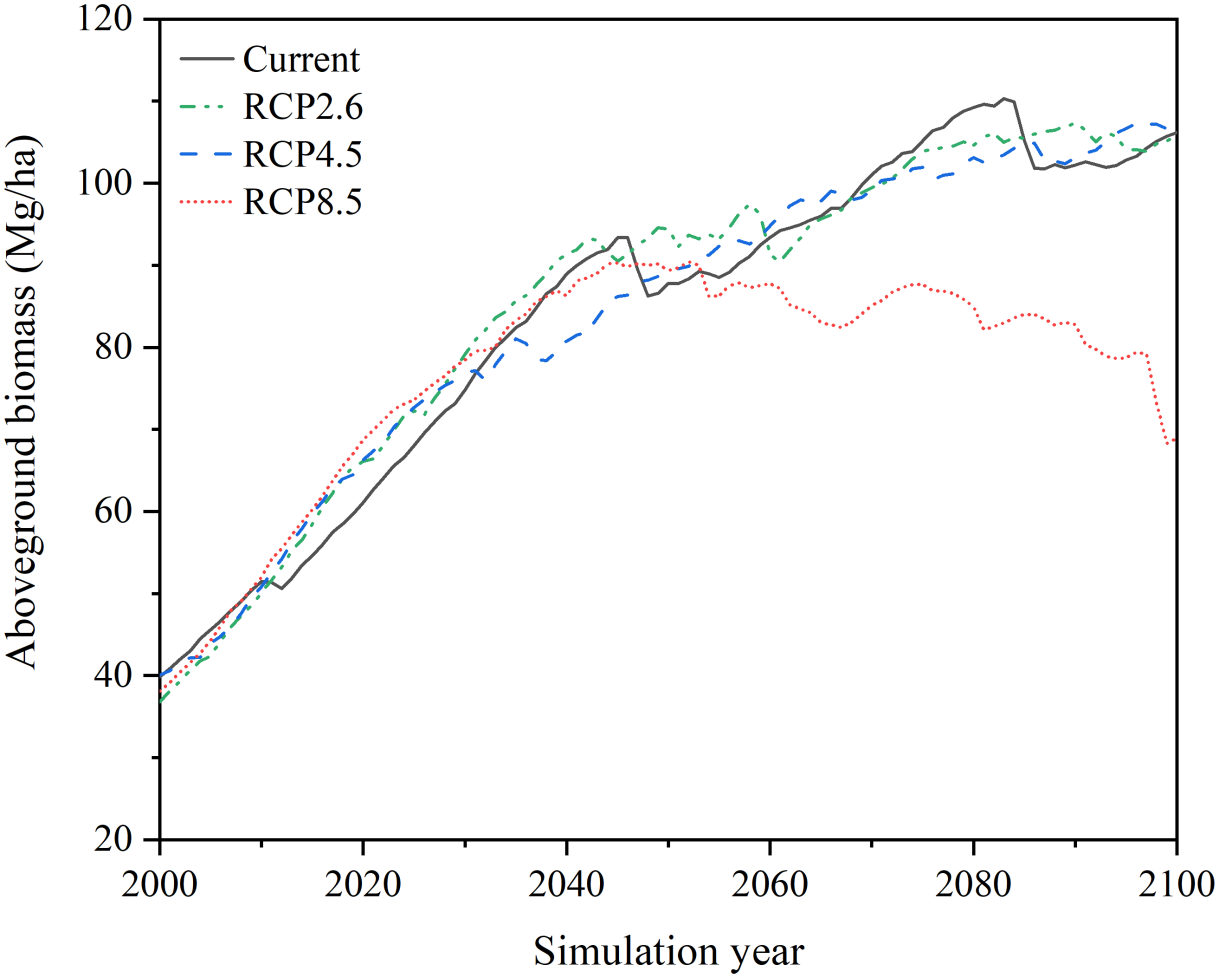
Dynamics of total aboveground biomass (AGB, Mg/ha) of 17 tree species in the forest region of Northeast China under four different climate scenarios (current, RCP2.6, RCP4.5, and RCP8.5) during the simulation years 2000–2100.

The simulation results generated by LINKAGES 3.0 indicate that within the current, RCP2.6, and RCP4.5 climate scenarios, the total aboveground biomass of 17 tree species in the forest region of Northeast China showed a gradual upward trend over the next 100 years. However, for the RCP8.5 climate warming scenario, an initially increasing and then decreasing trend was observed for the total biomass of 17 tree species (**Figure 6**). Specifically, from the early 21st century to the mid-21st century, limited differences were observed in the total aboveground biomass of the 17 tree species simulated under the RCP8.5 scenario relative to that within the current, RCP2.6, and RCP4.5 climate scenarios, and all scenarios showed an upward trend. However, from the mid-21st century to the end-21st century, the total aboveground biomass simulated under the RCP8.5 climate scenario exhibited a declining trend, whereas that projected within the current, RCP2.6, and RCP4.5 climate scenarios continued to rise gradually. As a result, the gap total aboveground biomass values increased between the RCP8.5 scenario and the other three climate scenarios increased. Specifically, by 2100, the RCP8.5 scenario resulted in a simulated total aboveground biomass of only 69.28 Mg/ha, whereas the current, RCP2.6, and RCP4.5 climate scenarios produced significantly high values of 106.16 Mg/ha, 104.70 Mg/ha, and 106.63 Mg/ha, respectively.

**Figure 7:**
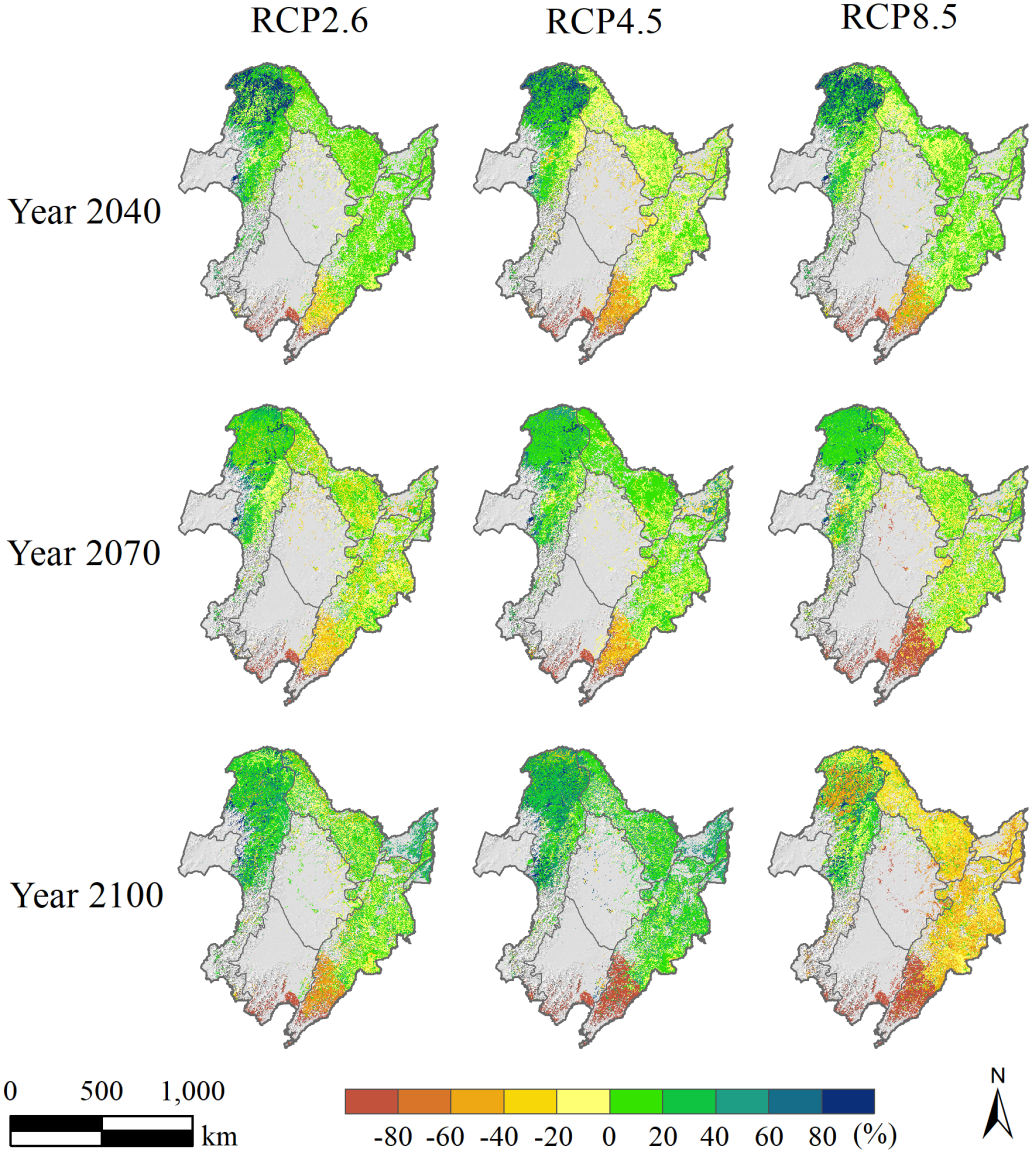
Spatial distribution of total aboveground biomass percentage variations (units: %) in different simulation years (2040, 2070, and 2100) within the three climate warming scenarios (RCP2.6, RCP4.5, and RCP8.5) relative to the current climate scenario in the forest region of Northeast China.

The simulations revealed clear spatial and temporal differences in the total aboveground biomass of 17 tree species in the forest region of Northeast China under various climate warming scenarios (**Figure 7**). In 2040, across three climate warming scenarios (RCP2.6, RCP4.5, and RCP8.5) relative to the current climate scenario, the total aboveground biomass within the investigated region was higher in the GKM, especially in the deciduous and coniferous forest regions situated northern GKM, whereas generally lower values were observed in the southern part of the CBM. Moreover, in the LKM and northern parts of the CBM, the differences between the simulated total aboveground biomass under the three climate scenarios and those simulated under the current scenario were not significant, with all values falling within the range of −20% to 20%. As the simulation progressed, the spatial variation in the total aboveground biomass decreased by 2070, although the difference in the aboveground biomass in the southern part of the CBM remained the most significant. By 2100, the total aboveground biomass in the majority of forest regions (excluding the southern part of the CBM) exhibited a notable increase in simulations within the RCP2.6 and RCP4.5 climate scenarios, with the simulated values significantly surpassing those of the current climate scenario. In contrast, the simulated total aboveground biomass under RCP8.5 was lower than the simulated values under the current climate scenario in most regions, except for some areas in the GKM.

**Figure 8:**
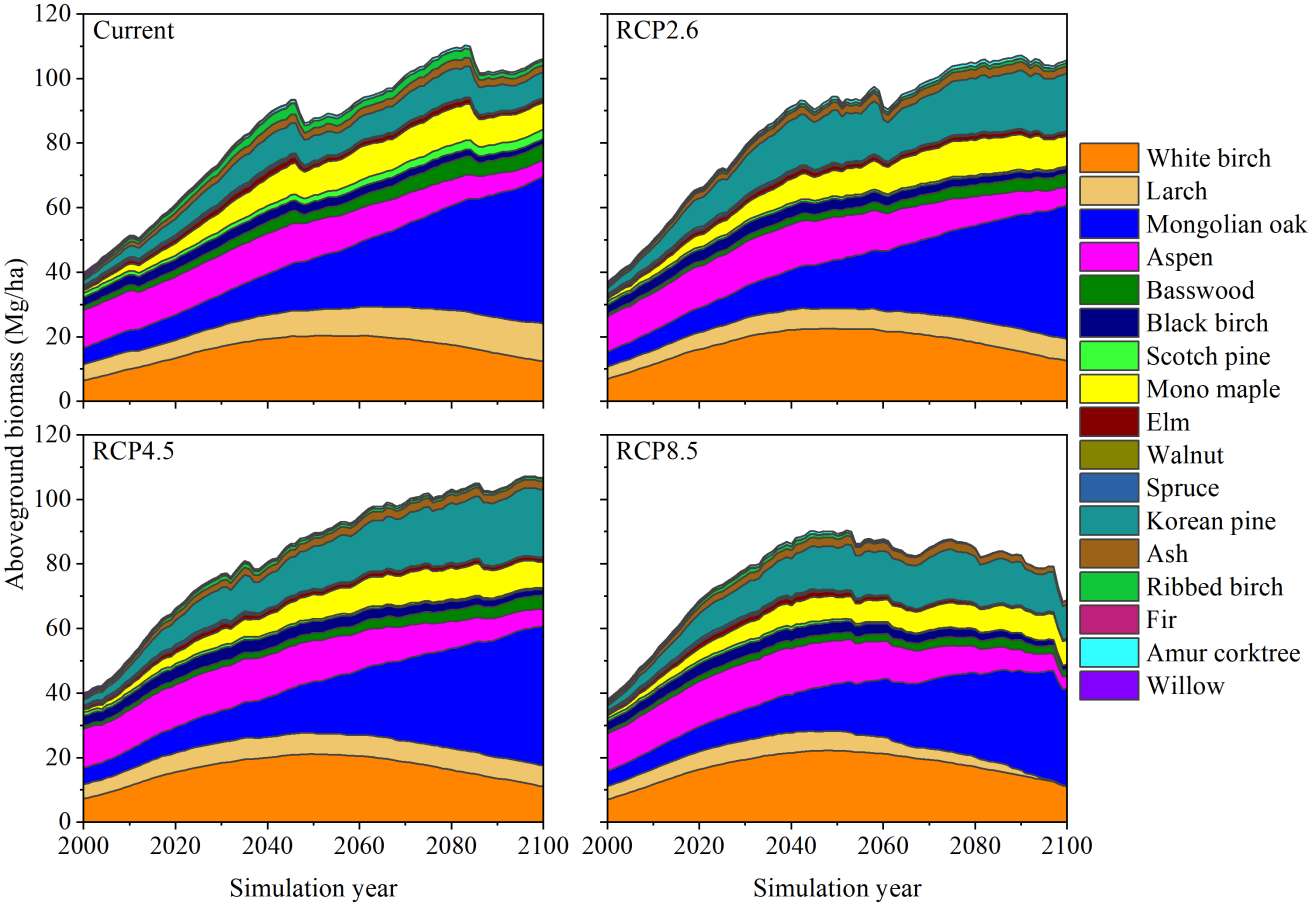
Dynamic simulations of total aboveground biomass (AGB, Mg/ha) composition of 17 tree species in the forest region of Northeast China across four climate scenarios (current, RCP2.6, RCP4.5, and RCP8.5) during the simulation years 2000–2100.

Throughout the simulation period (2000–2100), the tree species composition in the forest region of Northeast China underwent dynamic changes within different climate scenarios (**Figure 8**). Overall, within the next 100 years, encompassing the current and three climate warming scenarios (RCP2.6, RCP4.5, and RCP8.5), the tree species composition will remain relatively rich. An analysis of the response of the individual tree species to climate warming indicated that White birch and Aspen displayed an initial increase and subsequent decrease in aboveground biomass across the four climate scenarios, Mongolian oak consistently showed an increased growth advantage throughout the simulation period and presented the largest proportion among the tree species in the forest region of Northeast China. The proportion of Mono maple also increased gradually but showed a slight decline in later stages. The proportion of Basswood remained consistent across the climate scenarios and gradually increased over time, with the current scenario producing a slightly higher value. Similar to Aspen, Black birch also exhibited an initially slow increase in aboveground biomass, followed by a significant decline. Notably, under the RCP4.5 scenario, Black birch had the highest simulated aboveground biomass. Elm and Ash both displayed an increasing-then-decreasing trend, with Elm declining earlier and more significantly. Other tree species, such as Ribbed birch, Walnut and Amur corktrees, exhibited relatively smaller proportions and insignificant variations in terms of aboveground biomass. With respect to coniferous tree species, the aboveground biomass of Korean pine significantly increased across all climate scenarios, particularly under three climate warming scenarios. Larch demonstrated a gradual upward trend within the current, RCP2.6, and RCP4.5 scenarios but a decline within the RCP8.5 scenario, ultimately reaching the near-eradication stage. The aboveground biomass of Scotch pine increased in the current scenario but decreased under the three RCPs scenarios. Spruce and Fir consistently exhibited low aboveground biomass, with the maximum aboveground biomass of Fir presenting values below 0.2 Mg/ha.

### 3.4 Tree species distribution

Climate, succession (year), and their interactions had significant impacts on the biomass changes and region-wide distribution of 17 tree species from 2000 to 2100, whereas their effects on the species percent occurrence differed markedly (**Table 1**). For species occurrence, the impact of succession was minimal and ranged from 2% to 16%, whereas climate change and the interaction between climate change and succession had a major impact on the variation in species occurrence, explaining 20–70% of the variance. For most tree species, the interactive effect between climate change and succession accounted for 43–72% of the species occurrence changes, while climate change explained 30–50%. The effect of the interaction on species occurrence was more pronounced than the individual effects of climate change and succession.

**Table 1:** Assessment of explained variability and statistical significance regarding the impacts of climate change, year, and the interaction of climate change × year on the region-wide percent occurrence (%) of 17 tree species in Northeast China, as predicted by the LINKAGES 3.0 model from 2000 to 2100.

Species name	Climate change		Year		Climate change × year	
	Variation explained (%)	*P*	Variation explained (%)	*P*	Variation explained (%)	*P*
White birch	29.55	<0.001	8.57	<0.001	61.88	<0.001
Larch	31.28	<0.001	14.66	<0.001	54.07	<0.001
Mongolian oak	23.67	<0.001	12.75	<0.001	63.58	<0.001
Aspen	44.60	<0.001	4.38	<0.001	51.02	<0.001
Basswood	51.24	<0.001	5.42	<0.001	43.35	<0.001
Black birch	47.60	<0.001	4.63	<0.001	47.77	<0.001
Scotch pine	41.27	<0.001	11.34	<0.001	47.39	<0.001
Mono maple	25.03	<0.001	5.60	<0.001	69.38	<0.001
Elm	35.67	<0.001	5.25	<0.001	59.08	<0.001
Walnut	28.81	<0.001	8.39	<0.001	62.81	<0.001
Spruce	21.32	<0.001	6.00	<0.001	72.69	<0.001
Korean pine	48.19	<0.001	7.17	<0.001	44.64	<0.001
Ash	36.74	<0.001	4.54	<0.001	58.72	<0.001
Ribbed birch	68.74	<0.001	14.18	<0.001	17.08	<0.001
Fir	59.08	<0.001	16.53	<0.001	24.39	<0.001
Amur corktree	32.59	<0.001	7.92	<0.001	59.49	<0.001
Willow	60.67	<0.001	2.46	<0.001	36.87	<0.001

**Figure 9:**
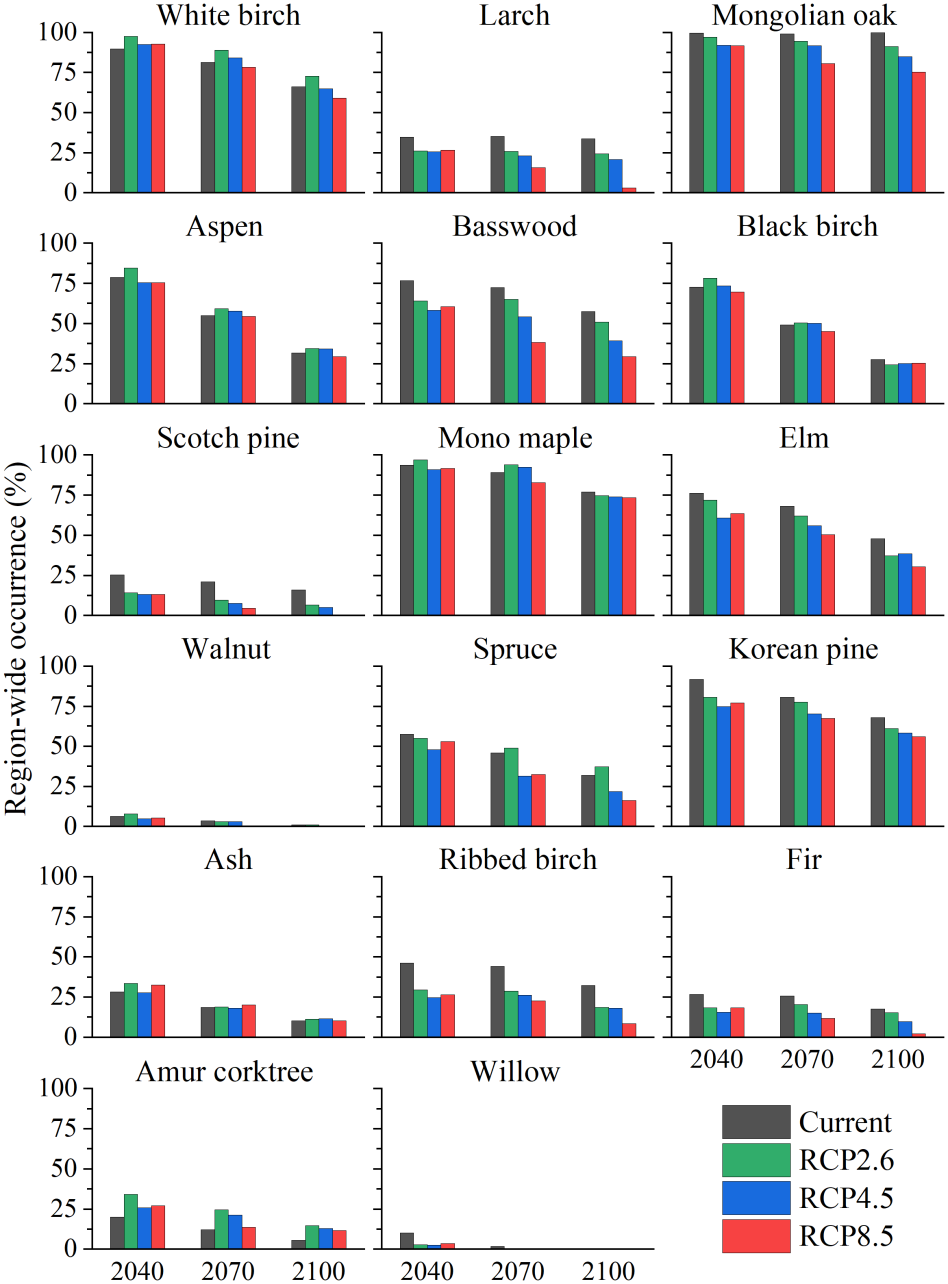
Region-wide occurrences (%) for 17 tree species across the current, RCP2.6, RCP4.5, and RCP8.5 climate scenarios for 2040, 2070, and 2100 in the forest region of Northeast China.

The occurrence of 17 tree species over time in Northeast China under four climate scenarios revealed that the climate and succession dynamics exerted a substantial effect on the potential distribution of these species (**Figure 9**). Among the numerous broad-leaved tree species, including White birch, Aspen, Black birch, Mono maple, Walnut, Ash, and Amur corktree, the simulated occurrences under different climate scenarios were relatively similar, although they all decreased with the progression of succession years (2040, 2070, and 2100). However, for the coniferous tree species of Scotch pine, Korean pine and Fir, as well as broad-leaved tree species of Basswood, Elm, Ribbed birch, and Willow, the occurrences generally decreased with the succession year and distinct differences occurred among various climate scenarios. Larch and Mongolian oak exhibited significant differences in occurrence across different scenarios, with current > RCP2.6 > RCP4.5 > RCP8.5; however, their occurrence remained relatively consistent across different years. Compared with other tree species, Spruce lacks a clear regularity in occurrence pattern but still experienced a decrease in occurrence with the passage of time, which was largely due to the significant impact of the interaction of climate and succession, which accounted for 72.69% of the changes. Taken together, the occurrence of nearly all tree species experienced a marked reduction as succession proceeded, and the differences in occurrence manifested across different climate scenarios and tree species.

**Figure 10:**
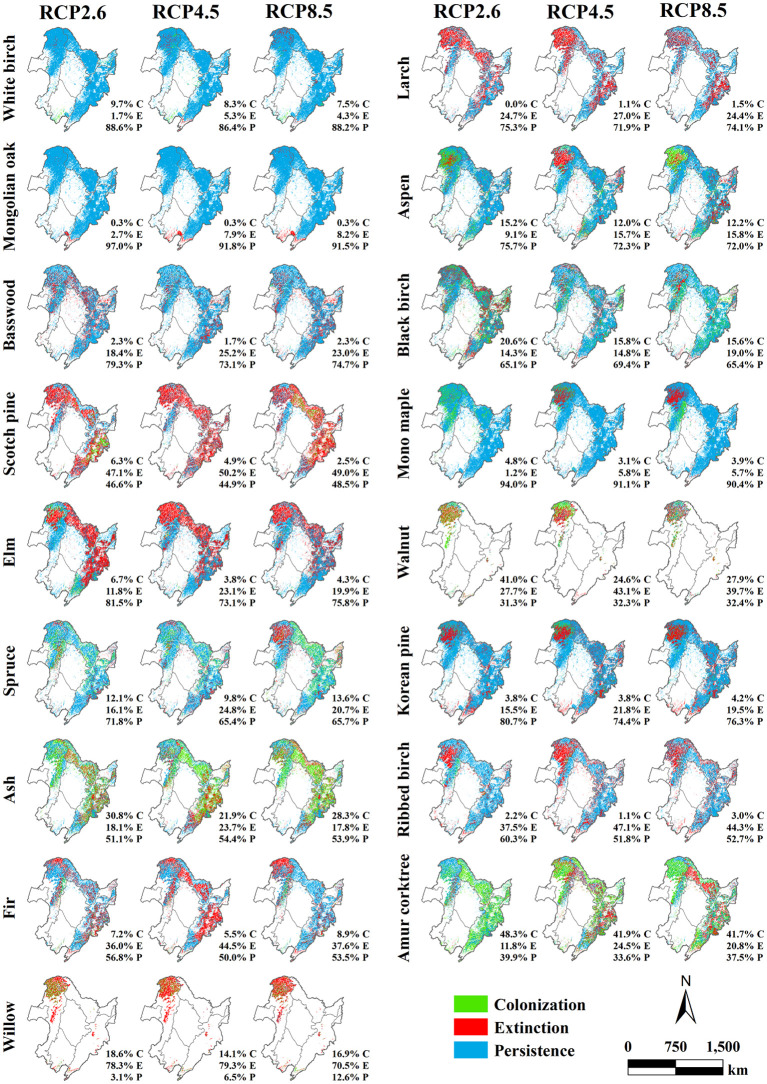
Spatial distribution and proportions of the predicted colonization (in green), extinction (in red), and persistence (in blue) for 17 tree species across three climate warming scenarios (RCP2.6, RCP4.5, and RCP8.5) relative to the current climate scenario in 2040 in the forest region of Northeast China.

**Figure 11:**
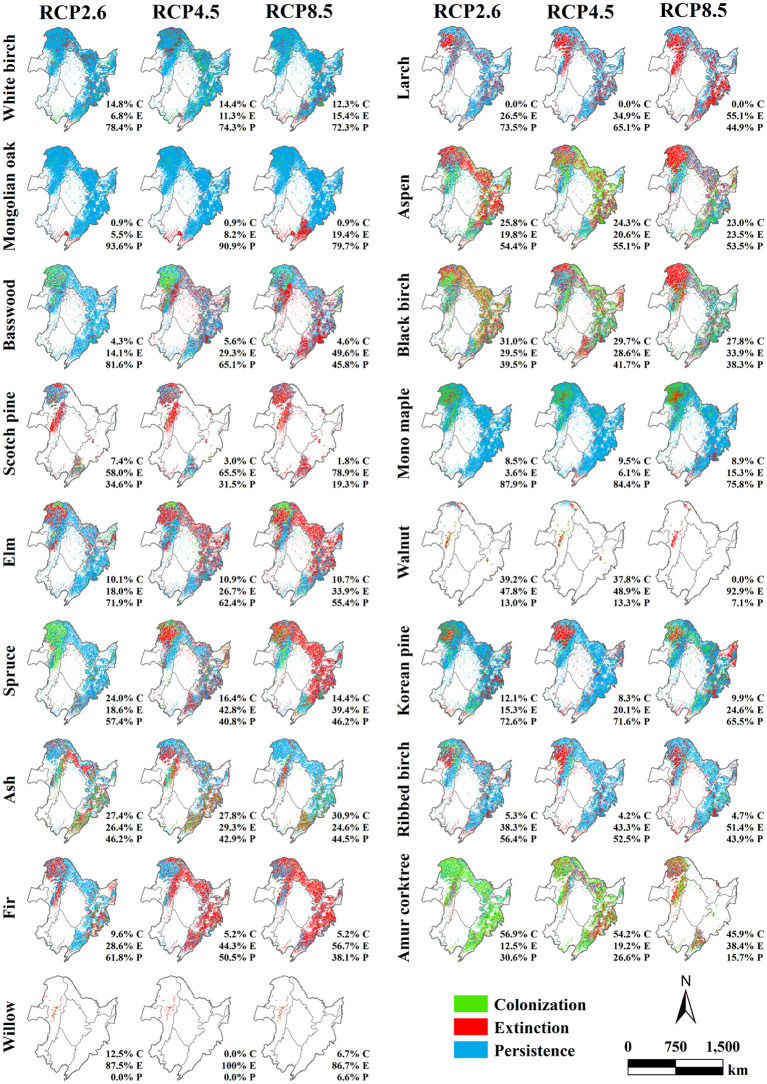
Spatial distribution and proportions of the predicted colonization (in green), extinction (in red), and persistence (in blue) for 17 tree species across three climate warming scenarios (RCP2.6, RCP4.5, and RCP8.5) relative to the current climate scenario in 2070 in the forest region of Northeast China.

**Figure 12:**
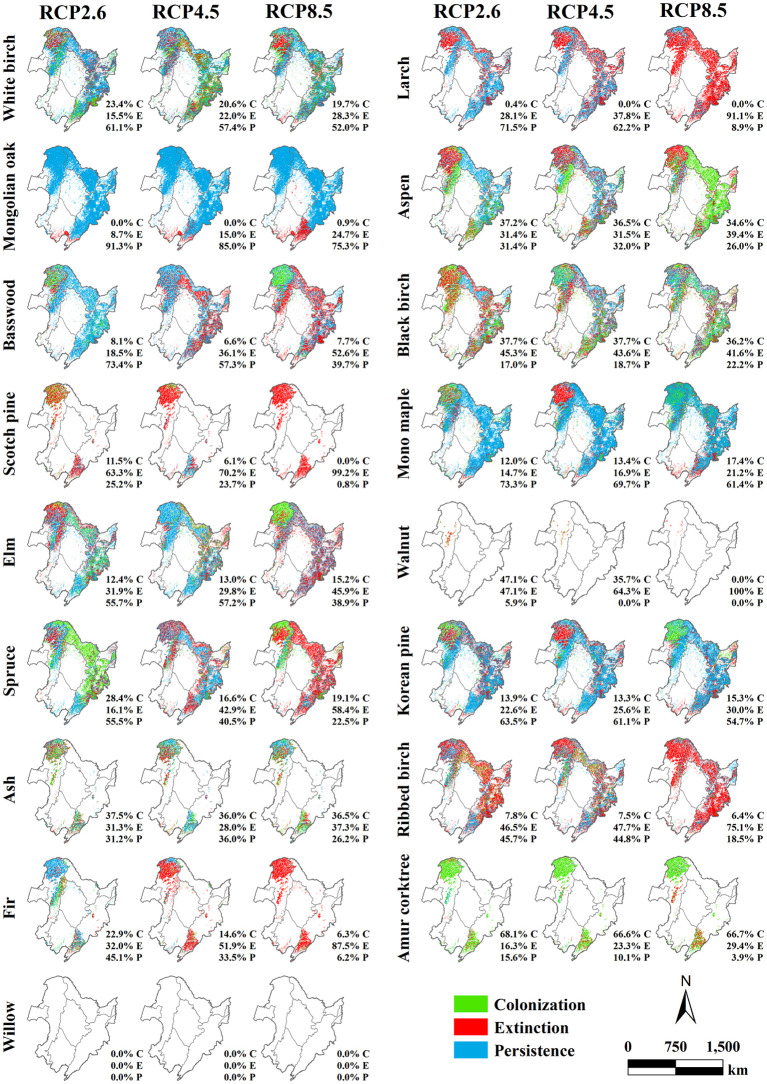
Spatial distribution and proportions of the predicted colonization (in green), extinction (in red), and persistence (in blue) for 17 tree species across three climate warming scenarios (RCP2.6, RCP4.5, and RCP8.5) relative to the current climate scenario in 2100 in the forest region of Northeast China.

Our research further analyzed the distribution and proportion changes of tree species within the three climate scenarios (RCP2.6, RCP4.5, and RCP8.5) in 2040, 2070, and 2100 compared with that within the current climate scenario (**Figures 10**–**12**). In 2040 (**Figure 10**), significant differences were not observed in the simulated biomass among the different climate scenarios, and most tree species exhibited high persistence proportions. For White birch, Mongolian oak, and Mono maple, clear persistence was observed in the forest region of Northeast China, with over 90% of the pixels exhibiting such a potential distribution. This finding suggested that the survival status of these three tree species was less affected by climate change and succession. Larch, Basswood, Elm, and Korean pine also showed high persistence rates (approximately 70%), although their extinction rates were also prominent, accounting for 20%. Similarly, Aspen, Black birch, and Spruce had a persistence rate of approximately 70% but also showed 15% colonization and 15% extinction rates. By 2070 (**Figure 11**), simulations predicted a noteworthy disparity in biomass for RCP8.5 and the other two climate warming scenarios (RCP2.6 and RCP4.5) in contrast to that of the current scenario, highlighting the influence of climate change on the potential tree species distribution. The results indicate a general decline in persistence rates relative to 2040 and an increase in colonization and extinction rates.

By 2100 (**Figure 12**), the effects of climate change on the potential distribution of various tree species will intensify, as evidenced by the significant differences in the spatial and proportional variety of simulated biomass under different climatic conditions. For instance, despite the dominance of Larch persistence in most areas within the RCP2.6 and RCP4.5 scenarios, the extinction rate of Larch exceeded 90% within RCP8.5. Similarly, species such as Basswood, Elm, Spruce, Ribbed birch, and Fir, also exhibited high extinction rates under RCP8.5, with many areas showing persistence within the RCP2.6 and RCP4.5 scenarios transitioning to extinction within RCP8.5. However, Aspen, Black birch, and Ash exhibited a relatively balanced ratio of persistence, extinction, and colonization, with a scattered spatial distribution lacking any clear patterns. Notably, several tree species showed similar potential distribution characteristics across all three climate scenarios, such as White birch, Mongolian oak, Mono maple, and Korean pine, all of which exhibited evident persistence in 2100 compared to the current climatic conditions, while Scotch pine and Amur corktree demonstrated extinction and colonization distribution characteristics, respectively. Moreover, Walnut and Willow showed only a sporadic distribution in the forest region of Northeast China, with no significant variations in their potential distribution characteristics.

## 4 Discussion

### 4.1 Spatiotemporal variations of aboveground biomass

The time pattern predicted by the LINKAGES 3.0 model showed a continuous increase in total aboveground biomass in the forest region of Northeast China throughout the first half of the 21st century within the four different climate scenarios. This positive trend can be attributed to the favorable influence of climate warming on vegetation growth in this region, with elevated temperatures and increasing precipitation extending tree-growing season and promoting overall vegetation development (Jeong et al., 2011; Qiang et al., 2016). Nonetheless, under RCP8.5 climate scenario, the simulated total aboveground biomass showed a decreasing trend from the mid-21st century until the end-21st century, which was inconsistent with the other three climate scenarios. This may be due to the excessively high annual Tmin and Tmax in the forest region of Northeast China in the mid- to long-term simulation under RCP8.5 scenario (**Figure 3**). These temperatures have already surpassed the threshold of suitable temperatures for growth in multiple tree species, which has led to the regression of numerous species and ultimately resulted in the reduction of total aboveground biomass (Fu and Sun, 2022).

Our research revealed pronounced spatial heterogeneity in the total aboveground biomass across the study area under all four climate scenarios. Specifically, the southern part of the CBM had the lowest aboveground biomass, while the northern part of the GKM had a relatively higher aboveground biomass. The total aboveground biomass in the southern CBM was the lowest among all regions within the three climate warming scenarios and significantly lower compared to the simulated values under the current climate. This discrepancy is likely due to a significant reduction (greater than 20%, **Figure 4**) in annual precipitation in the southern CBM, which has resulted in water shortages and droughts that have negatively affected the growth of multiple tree species and led to reduced biomass. Conversely, the higher biomass observed in the northern GKM may be ascribed to relatively notable enhancements in temperature and precipitation (**Figures 4**, **5**). The increase in aboveground biomass can be regarded as a favorable consequence of regional climate change; however, excessive warming is associated with a decrease in forest growth. In the forest region of Northeast China, warmer and drier climates have caused a decline in biomass within coniferous-broadleaf mixed forests, specifically in the southern portion of the CBM and high-elevation regions.

### 4.2 Effects of climate change on tree species composition and distribution

Climate change and succession exert notable influences on the potential distribution of tree species, particularly regarding their occurrence, with the influence of climate change surpasses that of succession. We found that the occurrences of most tree species in the forest region of Northeast China have significantly decreased due to climate change and successional dynamics, highlighting the adverse impacts of succession and climate warming on tree species presence. However, by 2100, minimal changes in the occurrences of Larch and Mongolian oak were found, which can be interpreted as a lagged response of these trees to climate change, with the distribution changes of some tree species potentially requiring several centuries to become apparent. Our method emphasizes the importance of considering species demography (growth, birth, death, and dispersal) when predicting changes in tree species distribution under climate change scenarios.

Climate warming has significantly impacted the distribution and composition of tree species in the forest region of Northeast China. Our research indicates that the distributions of Mono Maple, Basswood, and Elm demonstrated significant differences under varying magnitudes of climate warming and climate scenarios, primarily because of their high sensitivity to temperature fluctuations (Adams and Kolb, 2005; Joséphine, 2022). According to Zhou et al. (2007), the critical high-temperature threshold for Mono maple survival is approximately 6°C, while Basswood struggles to adapt when the temperature exceeds 4°C. In contrast, a temperature increase exceeding 4°C decreased the occurrence of Elm, although the change was less significant compared to that of Basswood. Consistent with these findings, our simulated results demonstrated that in response to a rise of over 4°C in annual Tmin in Northeast China, the potential distribution area of Mono maple exhibited a slight northward migration, while that of Basswood experienced a rapid reduction. Meanwhile, the potential distribution area of Elm remained relatively stable and showed only a minimal decrease.

Our predictions generally align with those of the ecological niche model (Gilani et al., 2020), biophysical process models (Morin et al., 2008), landscape models (Wang et al., 2018a, b), and process-landscape coupled models (Wang et al., 2017), indicating that coniferous tree species in mid to high latitudes (e.g., Larch, Scotch pine, Spruce, and Fir) are likely to face a substantial decline under severe climate warming scenarios, while broad-leaved tree species (e.g., White birch, Mono maple, and Mongolian oak) may experience an increase in their proportion. This is mainly because Mongolian oak, White birch, and Mono maple are strongly adaptable and drought-tolerant species with good sprouting ability, heliophilicity, and fire tolerance (Wang, 2006; Ma and Lei, 2015). Even under the RCP8.5 climate scenario, these species have been observed to undergo rapid adaptation to adverse environmental conditions, thus exhibiting greater production potential. Larch, Scotch pine, Spruce, and Fir can adapt to cold environments; however, their growth and distribution encounter major challenges owing to the escalating impacts of climate warming (Sun et al., 2010; Bosela et al., 2018). The simulated outcomes from our study suggest that under intensified climate warming, especially within the RCP8.5 scenario, the annual Tmin in Northeast China is projected to increase by up to 4.39°C, which may have already met or exceeded the adaptation threshold for the tree species mentioned above. Consequently, these species may lose suitable living conditions, fail to grow or regenerate, experience rapid shrinkage, or even disappear from their potential distribution areas. Additionally, our approach considers fundamental ecosystem processes and can adjust climate parameters to predict the spatial shift towards northward migration for most temperate broad-leaved tree species and Korean pine. This outcome is in line with studies conducted at the same latitude in the northeastern United States (Louis et al., 2008; Wang and Martin, 2017).

### 4.3 Significance and limitations

Previous studies have employed earlier versions of the LINKAGES model to investigate the dynamic changes in the forest region of Northeast China (Huang et al., 2022). The potential reactions of major tree species to climate warming in the LKM, as well as the simulation results of the key tree species within the same climate scenario in the GKM obtained in these studies, exhibit consistent with the findings of our study (Zhou et al., 2007; Zheng, 2011). Notably, the aforementioned studies have primarily focused on individual and sporadically distributed small forested areas. However, this study stands out as the inaugural examination encompassing the entire forest region of Northeast China as the research object and provides important theoretical support for forest management and decision-making. Our research indicates that tree species, such as Mongolian oak, White birch, and Korean pine, should be supported to bolster the adaptability and resilience of forests to climate warming. Conversely, tree species, such as Larch, Scotch pine, Ribbed birch, Spruce and Fir, might encounter difficulties in rapidly acclimating to climate scenarios with significant temperature increases, and artificial migration may be required to facilitate forest adaptation.

The validation results using forest inventory data indicated that the simulated results of the LINKAGES 3.0 model were higher than the actual corresponding total biomass in the surveyed plots. This phenomenon may be attributed to the model not considering the impacts of disturbances, such as windstorms, fires, pests, and diseases, on tree mortality, growth, and establishment, which have been documented to significantly affect forest growth (Yu et al., 2011). The forest region of Northeast China is characterized by its dense and complex vegetation cover, often extending into inaccessible areas not reachable by human effort. Despite meticulous considerations of plot representativeness, tree species typicality, and location accessibility during field surveys, achieving comprehensive coverage of the entire region remains challenging. In subsequent research, we aim to employ strategic and targeted sampling methods to select additional representative sampling points and reassess current plots, striving to ensure a more uniform and comprehensive spatial distribution of tree species and enhance the reliability of our results. Although the model incorporates the direct impact of drought on tree growth, it fails to consider landscape disturbances, seed dispersal process, forest management, or spatial ecological processes, resulting in potential biases. Meanwhile, the model does not differentiate between protected and managed forests and non-protected open areas. One potential solution to tackle these limitations is by integrating LINKAGES 3.0 with forest landscape models, like LANDIS PRO (Huang et al., 2017, 2018). In summary, predictions generated by the LINKAGES 3.0 model only depicted spatial locations suitable for tree growth (i.e., potential niche), and caution should be exercised when extrapolating these predictions to represent the actual distribution areas of tree species.

## 5 Conclusion

This study employed the forest ecosystem model LINKAGES 3.0, which fully accounts for the influence of interspecies competition on tree growth, to simulate the forest composition dynamics and potential spatial distribution of 17 composite tree species in the forest region of Northeast China under different climate scenarios (current, RCP2.6, RCP4.5, and RCP8.5). The findings suggested that over the next 100 years, climate warming is projected to exert a predominantly favorable impact on aboveground biomass, and significant spatial heterogeneity in the magnitude and direction of biomass change will occur. Global warming is expected to result in a decline in the proportion of coniferous species and an increase in the proportion of broad-leaved species. Cold-temperate conifers will gradually shift to warm-temperate broad-leaved tree species, thus forming a mixed forest dominated by Mongolian oak, white birch, mono maple, and Korean pine. Under the warming scenario, the potential distributions of many tree species are anticipated to migrate northward, and the extent of migration is expected to be more pronounced with greater warming. Moreover, with further rapid temperature increases, certain species may exhibit limited acclimation capabilities within a short time frame, resulting in gradual deterioration or even extinction. This study provides data to assist policymakers in comprehending tree species variations and their potential distributions, thereby empowering them to effectively address climate change challenges through prudent and adaptive forest management measures.

## Data availability statement

The original contributions presented in the study are included in the article/**Supplementary Material**. Further inquiries can be directed to the corresponding author.

## Author contributions

YF: Conceptualization, Funding acquisition, Methodology, Project administration, Supervision, Writing – review & editing. CL: Data curation, Visualization, Writing – original draft. HH: Conceptualization, Supervision, Writing – review & editing. SW: Funding acquisition, Writing – review & editing. LW: Writing – review & editing. ZX: Methodology, Software, Writing – review & editing.
