# Supplementary material for: Assessing the impact of climate warming on tree species composition and distribution in the forest region of Northeast China
Source: Front Plant Sci. 2024 Jul 29;15:1430025. doi: 10.3389/fpls.2024.1430025 (PMC11317430; doi:10.3389/fpls.2024.1430025)
Supplement: Supplementary file 1 [file Table_1.docx]

Supplementary Material

Supplementary Table S1. Major species parameters of 17 tree species in the forest region of Northeast China input to LINKAGES 3.0 model.

| **Species** | **DMAX** | **DMIN** | **B2** | **B3** | **ITOL** | **AGEX** | **G** | **SWITCH** | **D3** | **FROST** | **TL** | **FWT** | **SLTA** | **SLTB** | **RTST** | **FRT** |
| --- | --- | --- | --- | --- | --- | --- | --- | --- | --- | --- | --- | --- | --- | --- | --- | --- |
| White birch | *3100* | 600 | 0.94 | 94.52 | 2 | 150 | 173.04 | FFFFT | 0.412 | -38 | 4 | 248 | 0.804 | 0.069 | 0.8 | 1 |
| Larch | *2000* | 400 | 0.32 | 60.27 | 1 | 300 | 88.92 | FFFFT | 0.424 | -38 | 12 | 440 | 0.804 | 0.069 | 1 | 1 |
| Mongolian oak | *3100* | 1000 | 0.32 | 60.27 | 1 | 300 | 90.92 | FTTTT | 0.514 | -33 | 9 | 440 | 0.904 | 0.095 | 1 | 1 |
| Aspen | *3000* | 800 | 0.65 | 78.77 | 2 | 125 | 148.02 | TTFFT | 0.333 | -34 | 7 | 248 | 0.804 | 0.069 | 0.5 | 1 |
| Basswood | *2400* | 800 | 0.39 | 67.36 | 1 | 300 | 88.65 | FTFFF | 0.233 | -33 | 2 | 440 | 0.814 | 0.078 | 1 | 1 |
| Black birch | *3100* | 600 | 0.74 | 74.52 | 2 | 150 | 115.67 | TTTFF | 0.489 | -35 | 4 | 248 | 0.804 | 0.069 | 0.8 | 1 |
| Scotch pine | *1900* | 700 | 0.36 | 59.08 | 2 | 250 | 81.0 | TTTFF | 0.6 | -58 | 12 | 440 | 0.804 | 0.069 | 1 | 2 |
| Mono maple | *3200* | 1000 | 0.51 | 62.10 | 1 | 200 | 109.15 | TTFFF | 0.233 | -32 | 2 | 440 | 0.814 | 0.078 | 1 | 1 |
| Elm | *2700* | 900 | 0.35 | 63.62 | 1 | 250 | 105.35 | FFFFF | 0.176 | -33 | 5 | 440 | 0.804 | 0.069 | 1 | 1 |
| Walnut | 2650 | 850 | 0.23 | 41.40 | 2 | 250 | 104.99 | TFFFF | 0.276 | -32 | 2 | 440 | 0.428 | 0.074 | 1 | 1 |
| Spruce | *2500* | 600 | 0.35 | 63.62 | 1 | 300 | 90.79 | FTFFF | 0.187 | -32 | 11 | 440 | 0.804 | 0.069 | 1 | 3 |
| Korean pine | *2800* | 1000 | 0.28 | 62.96 | 1 | 300 | 118.53 | FTFFF | 0.276 | -33 | 12 | 440 | 0.804 | 0.069 | 1 | 2 |
| Ash | *2800* | 1000 | 0.33 | 67.26 | 2 | 250 | 121.89 | TTFFF | 0.187 | -29 | 2 | 440 | 0.428 | 0.074 | 1 | 1 |
| Ribbed birch | *1900* | 700 | 0.35 | 63.62 | 1 | 250 | 106.35 | TTFFT | 0.187 | -35 | 4 | 440 | 0.428 | 0.074 | 1 | 1 |
| Fir | *1800* | 400 | 0.39 | 67.36 | 1 | 300 | 87.64 | FTFFF | 0.233 | -32 | 10 | 440 | 0.804 | 0.069 | 1 | 3 |
| Amur corktree | *3200* | 1000 | 0.22 | 43.43 | 2 | 250 | 90.48 | TFFFF | 0.233 | -32 | 8 | 440 | 0.428 | 0.074 | 1 | 1 |
| Willow | 1600 | 600 | 0.35 | 63.62 | 2 | 180 | 94.03 | TTFFT | 0.1 | -35 | 7 | 248 | 0.804 | 0.069 | 1 | 1 |

DMAX, maximum growing degree day required for each tree species (℃).

DMIN, minimum growing degree day required for each tree species (℃).

B2 and B3 are parameters for Richard growth equation.

ITOL, shade tolerance coefficient for tree species.

AGEX, lifespan for each tree species.

G, growth parameter.

SWITCH, update logic switch function.

D3, drought coefficient, i.e., the number of days during the growing season when the soil humidity can be below the wilting point/total growing season days.

FROST, minimum tolerable temperature for tree species, i.e., the minimum monthly temperature the tree species can withstand.

TL, litter type.

FWT, leaf weight per unit crown area (100g/m^2^).

SLTA and SLTB are forest canopy area conversion parameters.

RTST, root-shoot ratio for each species.

FRT, retention period of leaves.
